# The Positive Value of Rotation-Specific Guides for Resident Education and Patient Care: A Process Improvement Study

**DOI:** 10.7759/cureus.17262

**Published:** 2021-08-17

**Authors:** Michelle Coughlin, Dianna Ehlert

**Affiliations:** 1 General Surgery, Detroit Medical Center/Wayne State University, Detroit, USA; 2 Vascular Surgery, Detroit Medical Center, Detroit, USA

**Keywords:** resident, education, pocket-guide, root-cause-analysis, rotation-specific, quality improvement research

## Abstract

Introduction

Providing high-quality cost-effective patient care requires knowledge of treatment protocols as well as an understanding of the institution's procedures, e.g., what orders to place and how to effectively place them. Disseminating rotation-specific evidence-based practice and institutional policies in a timely manner to medical residents rotating onto a service can be challenging. We determined, by root cause analysis, that a solution was to use a structured guide containing rotation-specific information. The purpose of this study was to evaluate the effectiveness of a rotation-specific pocket reference guide to distribute evidence-based and rotation-specific practice information to medical residents rotating through an Academic Vascular Surgery service and to evaluate this tool’s ability to increase participants’ perception of comfort and efficacy, all of which can be linked to high-quality and cost-effective patient care.

Materials and methods

We conducted a prospective study at the Detroit Medical Center, a Michigan-based level one trauma hospital, from November 2020 through February 2021. The inclusion criteria included medical residents that were on the Vascular Surgery rotation in the given time frame and that agreed to take a pre-/post-evaluation. The evaluation consisted of a quantitative test and a qualitative questionnaire. A t-test was used to analyze pre- and post-question score averages.

Results

There was a significant improvement in quantifiable knowledge as participants’ scores increased on post-rotation testing scoring with an average of 88% post-rotation compared to 58% pre-rotation (p<0.01). Ancillary staff reported a decrease in incorrect orders, substantiating increased efficacy and inferring cost-effectiveness. Individuals evaluated post-rotation indicated the usefulness of the guide as an educational tool for the dissemination of evidence-based practice (p<0.01) and increased confidence in placing preoperative orders (p<0.01). This, coupled with a post-rotation increase in preference toward a written learning style, led to the additional conclusion that this guide would be a beneficial preparatory tool for future board examinations.

Conclusions

This study supports the implementation of rotation guides as a preparatory source used to improve the dissemination of rotation-specific information, which should increase resident efficacy, improve cost-effectiveness, and potentially improve future board examination scores. We recommend a chart review of specified metrics, e.g., incorrect order frequency and related operative delays, to show to what extent the cost-effectiveness and increase in high-quality patient care manifested.

## Introduction

As residents rotate through multiple services during their training, common obstacles are learning rotation-specific treatment information and learning administrative information for each service, such as contacts, documentation requirements, policies relevant for immediate and effective patient care, etc. To compound these problems, the ordering system at the institution studied used an order search feature that was specific to the first few letters typed into the search field. For example, the order set for contrast dye allergy was named “PGT”; typing keywords such as “contrast, dye, allergy” would yield no results.

The question then became how to best distribute the needed information to improve patient care and overall knowledge in a limited time frame. We determined the root cause was the lack of an easily available structured guide containing necessary rotation-specific information. 

The purpose of this study was to evaluate the effectiveness of a rotation-specific pocket reference guide to distribute evidence-based and rotation-specific practice information to medical residents rotating through an Academic Vascular Surgery service and to evaluate this tool’s ability to increase participants’ perception of comfort and efficacy, all of which can be linked to high-quality and cost-effective patient care. We evaluated the guide with two objectives: (1) to quantitatively measure the effectiveness of information dissemination using a multiple-choice pre-/post-test comparison and (2) to qualitatively evaluate the medical residents’ perceptions of increased efficacy, efficiency, and comfort, using a pre-/post-questionnaire. 

## Materials and methods

We created a 32-page Vascular Surgery Pocket Guide containing site-specific and evidence-based information in the following categories: Directory, Conferences and Meetings, Coverage & Follow-up, Order Sets, Boarding Cases, Allergies, Pre-operative (Pre-op)/ Post-op order sets, Antibiotics, Anticoagulation, Wounds, Fluids, Vascular Studies-Imaging Orders, Carotid and Renal Stenosis Classifications, Vascular Lab Diagnosis and Billing ICD 10 codes, Common Vascular Pathology {Abdominal Aortic Aneurysm ( AAA), Aortoiliac Disease, Aortoenteric Fistula, Carotid Stenosis/Stroke, Compartment Syndrome. Peripheral Artery Disease (PAD), Critical Limb Ischemia/ Vasopressor Related Ischemia, Venous disease/CEAP (Clinical-Etiology-Anatomy-Pathophysiology) Classifications}, Dialysis Access, Educational Resources, Anatomy and Pulses.

We conducted a prospective study of the use of a Vascular Surgery rotation-specific pocket reference guide at the Detroit Medical Center, a Michigan-based level one trauma teaching hospital, from November 2020 through February 2021. The institution’s Nursing Research Council deemed this a workflow improvement project and, therefore, did not require Internal Review Board approval. Participants were 12 medical residents on the Vascular Surgery service rotation. The testing was anonymous; no participant names were documented. As participation was voluntary, the inclusion criteria included medical residents that agreed to take a pre- and post-evaluation and were on the Vascular Surgery rotation in the given time frame. Residents who opted out or did not rotate on the vascular service in the given time frame were excluded. This yielded 12 pre-evaluation forms and eight post-evaluation forms (four residents were either unavailable or declined to fill out the post-evaluation forms).

Participants at the start of their rotation were given a test and questionnaire before being provided with the rotation guide. The 10-question test contained five service-specific (procedural) questions and five Vascular Surgery (knowledge) questions. The questionnaire gauged the comfort and efficacy of using core preoperative orders and the perceived value of rotation-specific guides using a scale of 1 through 5 with 1 = strongly disagree and 5 = strongly agree. Participants were then given the same test and questionnaire at the completion of the rotation. Participants' scores were analyzed using a two-sample t-test and Pearson’s correlation coefficient (p-value) with statistical significance given to those results with a p-value < 0.05 [1].

Participants were also asked to indicate their preferred education modality before and after the rotation, using correlations to the VARK model categories of visual, auditory, reading/writing, and kinesthetic learning styles [2]. The preferred learning method questionnaire categories were written instruction, watching others/doing or verbal instruction. Visual and kinesthetic were grouped as ‘watching others/doing’ on the questionnaire based on an industry teaching standard of ‘see one, do one, teach one’ and, in a surgical service, residents are often watching attendings and performing hands-on patient care in the operating room simultaneously.

## Results

**Figure 1:**
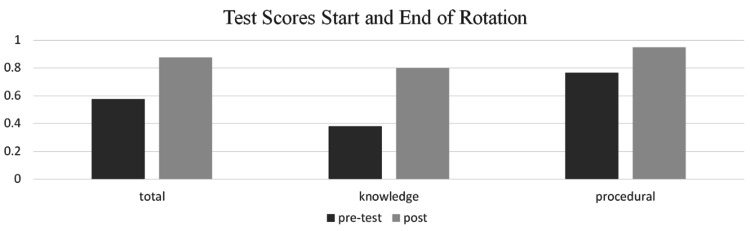
Test score comparison (total, knowledge-based questions, procedural-based questions). Knowledge: vascular knowledge questions. Procedural: institution policy questions.

There was significant improvement of quantifiable knowledge as participants’ scores increased on post-rotation testing with an average of 88% post-rotation compared to 58% pre-rotation (p<0.01). Test questions were representative of both knowledge, e.g., May-Thurner and CEAP (Clinical, Etiological, Anatomical and Pathophysiological) classification for venous disease, and procedural, e.g., contrast dye allergy prophylaxis and vascular imaging orders. There was a significant improvement in both tested categories: questions related to vascular knowledge versus institutional policy (p<0.01 and p=0.02, respectively, in Figure 1).

Qualitatively, increased confidence in placing pre-op orders (p<0.01) was reported with additional confirmation as the category of ordering pre-op antibiotics alone also indicated a statistically significant increase in perceived comfort level (p<0.01). Individuals evaluated post-rotation indicated they agreed to the usefulness of the guide as an educational tool for the dissemination of evidence-based practice (p<0.01). Scores pre- and post-rotation indicated a favorable outlook on guides for pre-op instructions and increasing efficiency. There was an increase but no statistically significant change in comfort caring for vascular patients in general (p=0.26). Residents’ perception that they could continue to improve in the patient care they provided remained favorable with an average questionnaire score of 4.33 pre-rotation and 4.38 post-rotation. Table 1 contains a complete numerical comparison of participants’ pre- and post-survey perceptions.

**Table 1:** Test and questionnaire pre- and post-rotation evaluation. *Average test scores with 1 = 100%. Questionnaire scale of 1-5 with 1 = strongly disagree and 5= strongly agree. Averages of all pre- and post-scores are represented in the table.

	Pre-rotation average	Post-rotation average	p-value
Test score*	0.58	0.88	<0.01
I am comfortable entering pre-op orders.	3.17	4.50	0.01
I know what pre-op antibiotics to order.	3.33	4.63	<0.01
Usefulness of rotation-specific guides.	3.25	4.50	<0.01
Rotation-specific guides disseminate evidence-based practice.	3.27	4.5	<0.01
I know what pre-op IV fluids to order.	3.33	4.88	0.05
I am comfortable ordering vascular studies.	3.00	3.88	0.09
I know contrast allergy pretreatment protocol.	2.67	3.63	0.40
I am comfortable treating vascular surgery patients.	3.09	4.00	0.26
I receive numerous communications regarding incorrect/missing orders e.g., from RN, etc.	2.58	2.62	0.57
I am anxious starting a new rotation.	3.33	3.12	0.74
Rotation-specific guides assist with the patient’s preoperative process.	4.17	4.25	0.87
Rotation-specific guides increase my efficiency.	4.25	4.38	0.97
Can improve in providing high-quality healthcare.	4.33	4.38	0.97

When residents were asked to report their preferred learning style, initially, 75% of participants indicated learning preference through watching others/doing (visual/kinetic), while written learning was only chosen as part of a multimodal style. Post-rotation, 25% of participants reported preference to learn with written instruction alone. 

## Discussion

While there were no other studies on the use of a pocket-sized reference guide, there were multiple studies on the use of other structured guides such as order sets and checklists. By advocacy of a systematic approach fostered by order sets and checklists, multiple studies have linked increased resident comfort [3], increased adherence to accepted protocols [4-6], improved prophylaxis outcomes [4], increased patient safety [4], cost-effectiveness [5,6], and increased perceived patient satisfaction to the use of a structured guideline [7].

O’Conner et al. found that order sets reduced the amount of inappropriate blood urea nitrogen laboratory test orders [4]. During our evaluation, ancillary staff reported a decrease in incorrect orders placed for vascular imaging, pre-op orders, and procedures; the responses of the medical residents to the question regarding the amount of correspondence received regarding incorrect orders showed only minimal awareness judging from little change in the pre- and post-questionnaire scoring (p=0.57). This can be explained by the fact that many of the incorrect orders were corrected by the Vascular Surgery Nurse Practitioner and never reached the medical residents. 

A decrease in incorrect orders would relate to a decrease in the cost for caring for a patient either with the decrease in extraneous testing, a decrease in operating room delays due to incomplete or missing orders or the decrease in time hospital staff needed to correct orders. We also found a reported increase in resident comfort in relation to ordering pre-op and antibiotic orders, which is broadly congruent with research conducted by Jaraback et al. [3] and Yu et al. [8]. 

Most participants initially indicated preferred learning through ‘watching others/doing’, yet the majority had reported an increased positive outlook on the contribution of written material. This was evidenced by the significantly increased score on the questionnaire question “Rotation specific guides disseminate evidence-based practice” and the post-rotation increase of professed preference toward using written materials to learn. Perhaps the questionnaire led, in part, to an introspection and re-evaluation of learning styles as was found in a classic study by Flemming and Mills [9].

Research correlating learning styles to the American Board of Surgery In-Training Examination (ABSITE) scores indicated that written learners have increased scores [10]. The success in the ABSITE testing is a predictor of success in board examinations. With an increase in written as the preferred learning style post-rotation coupled with the increase in test scores post-rotation, it is a reasonable conclusion that this guide would be a beneficial preparatory tool for future board examinations. 

An increase in test scores and service-related knowledge cannot be solely attributed to the guide as many opportunities for education were present during the Vascular Surgery rotation; however, research has proven that an organized and comprehensive guide leads to lower incorrect orders and less extraneous testing. This in turn translates to more cost-effective care. Our results congruently showed increased quantifiable knowledge and decreased incorrect orders. Therefore, it can be inferred that the Vascular Surgery guide increased cost-effectiveness and the quality of patient care. The cost-effectiveness and increase in high-quality patient care are only reasonable assumptions and further studies involving a chart review to show to which extent the supposition manifested are needed.

Other limitations to this study included the small participant population which negated statistical error corrections for competency obtained through any other source besides the guide. It would take years to gain higher numbers of participants with a rotation length of one month. Conversely, conducting this study in a short time frame worked in our favor as to not have the guide circulated before the pre-rotation testing and skew the results.

This quality improvement analysis indicated the positive value of a rotation-specific guide in the setting of a Vascular Surgery rotation. As this tool forwards the AGCME core competencies of patient care and procedural skills as well as systems-based practice [11], it could be modified for and be a benefit to medical residents on any medical service.

## Conclusions

This study supports the implementation of rotation guides as a preparatory source used to improve the dissemination of rotation-specific information, which should increase resident efficacy, improve cost-effectiveness, and potentially improve future board examination scores. We recommend a chart review of specified metrics, e.g., incorrect order frequency and related operative delays, to show to what extent the cost-effectiveness and increase in high-quality patient care manifested.
